# Supplementary figures and images for: Single-cell RNA profiling reveals classification and characteristics of mononuclear phagocytes in colorectal cancer
Source: PLoS Genet. 2024 Feb 26;20(2):e1011176. doi: 10.1371/journal.pgen.1011176 (PMC10919852; doi:10.1371/journal.pgen.1011176)

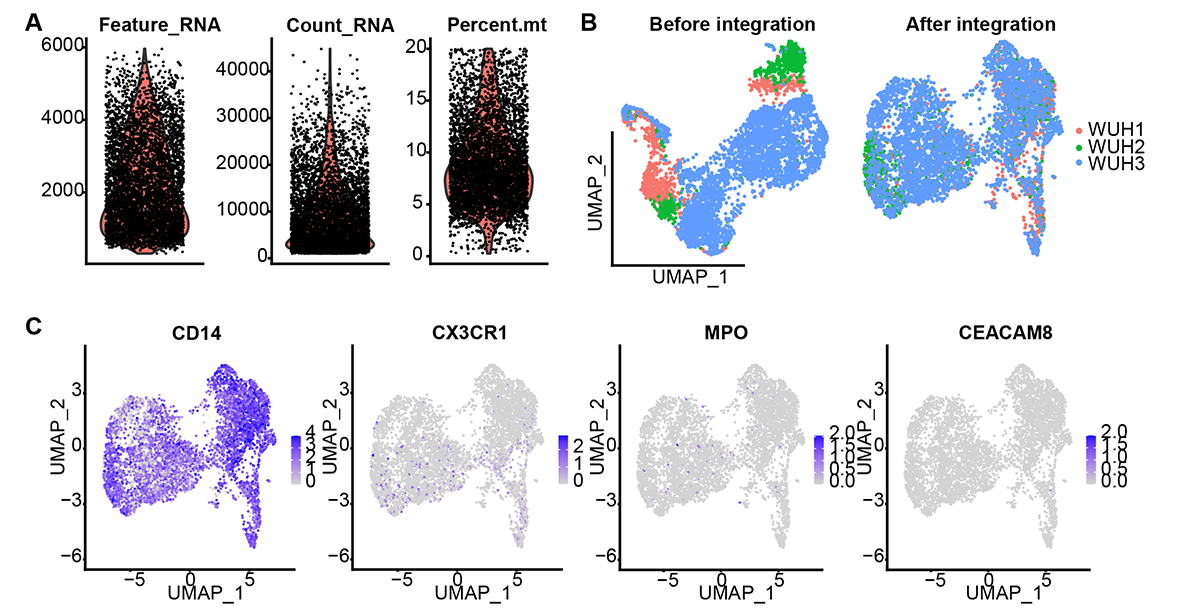

Supplement: S1 Fig — (A). Quality control of single-cell RNA sequence data. (B). UMAP plots of mononuclear phagocytes before integration (left) and after integration (right). Cells are colored by samples. (C). UMAP plots showed expression levels of marker genes of mononuclear phagocytes (CD14), intestinal dendritic cells (CX3CR1) and neutrophils (MPO and CEACAM8, also named CD66b). (TIF) [file pgen.1011176.s001.tif]

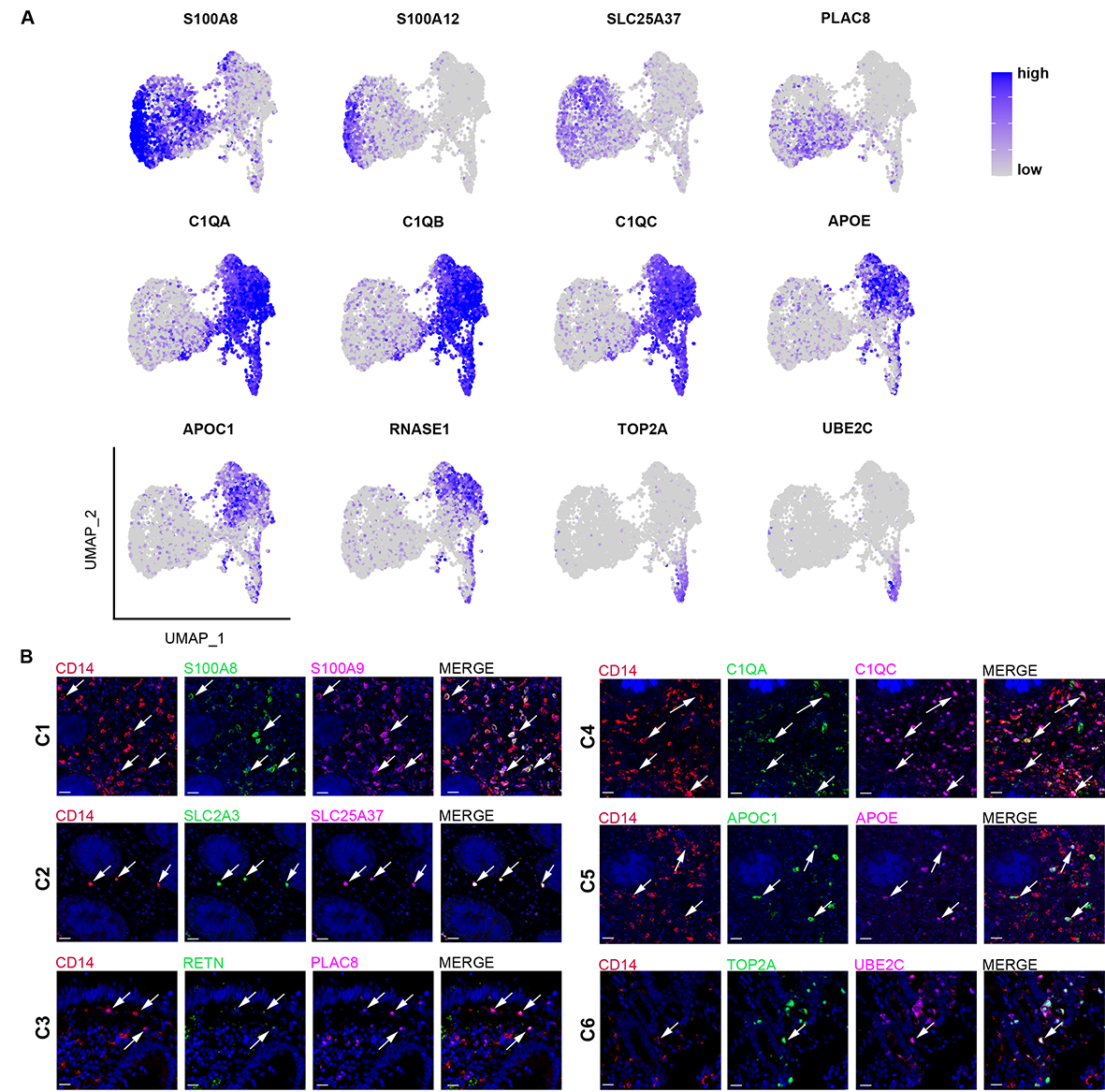

Supplement: S2 Fig — (A). UMAP plots showed expression levels of marker genes of each cluster. (B). Immunofluorescence staining of CD14 and marker genes of each cluster in CRC tissues confirmed the existence of six clusters. White arrows point cells of each cluster. Scale bars, 20 μm. (TIF) [file pgen.1011176.s002.tif]

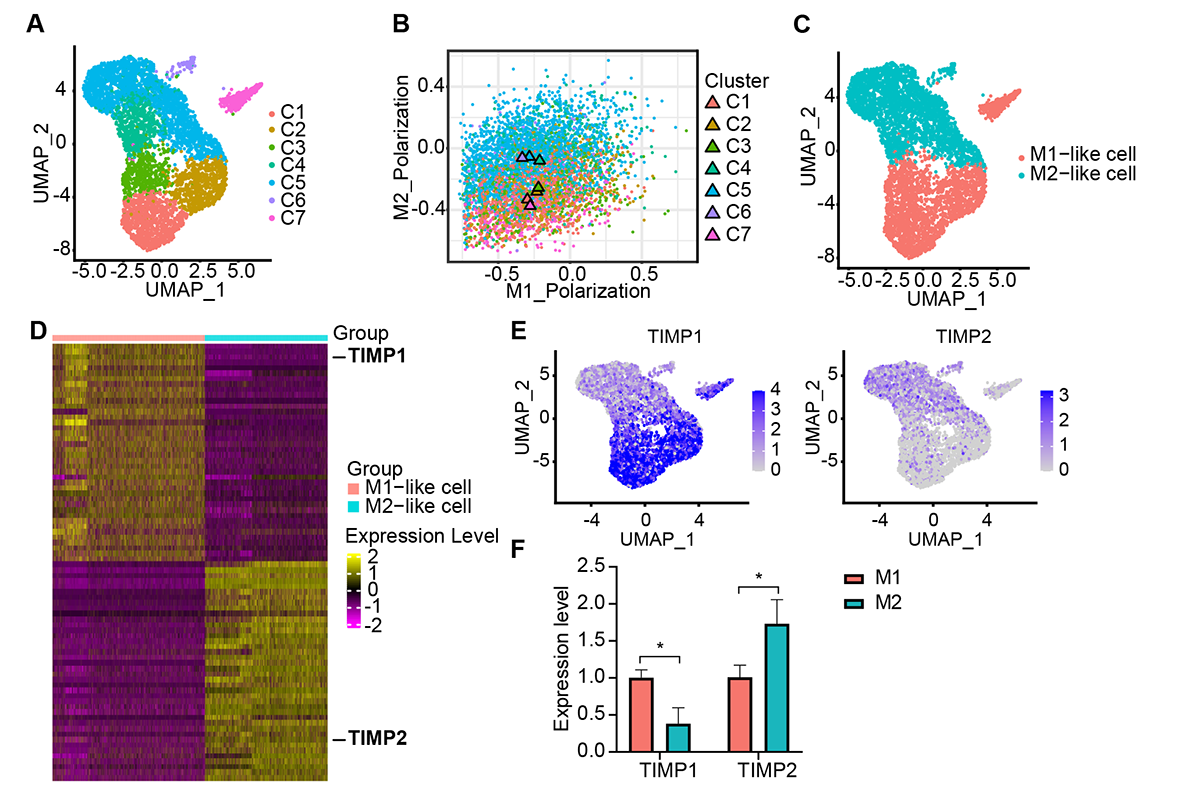

Supplement: S3 Fig — (A). UMAP plot of mononuclear phagocytes in GSE132465 CRC patients. Cells are colored by clusters. (B). Scatterplot showed M1 polarization and M2 polarization score of each cluster in GSE132465 CRC patients. The mean score of each cluster is represented by a color-coded triangle. (C). UMAP plot of mononuclear phagocytes in GSE132465 CRC patients, color-coded by M1-like and M2-like cell. (D). Heatmap displaying differential expression pattern of genes in M1-like and M2-like cells. (E). UMAP plots showed expression levels of TIMP1 and TIMP2 in GSE132465 CRC patients. (F). Expression levels of TIMP1 and TIMP2 in human peripheral blood-derived M1 and M2 cells (N = 3), *P < 0.05. The statistical significance was obtained by Student’s t-tests. (TIF) [file pgen.1011176.s003.tif]

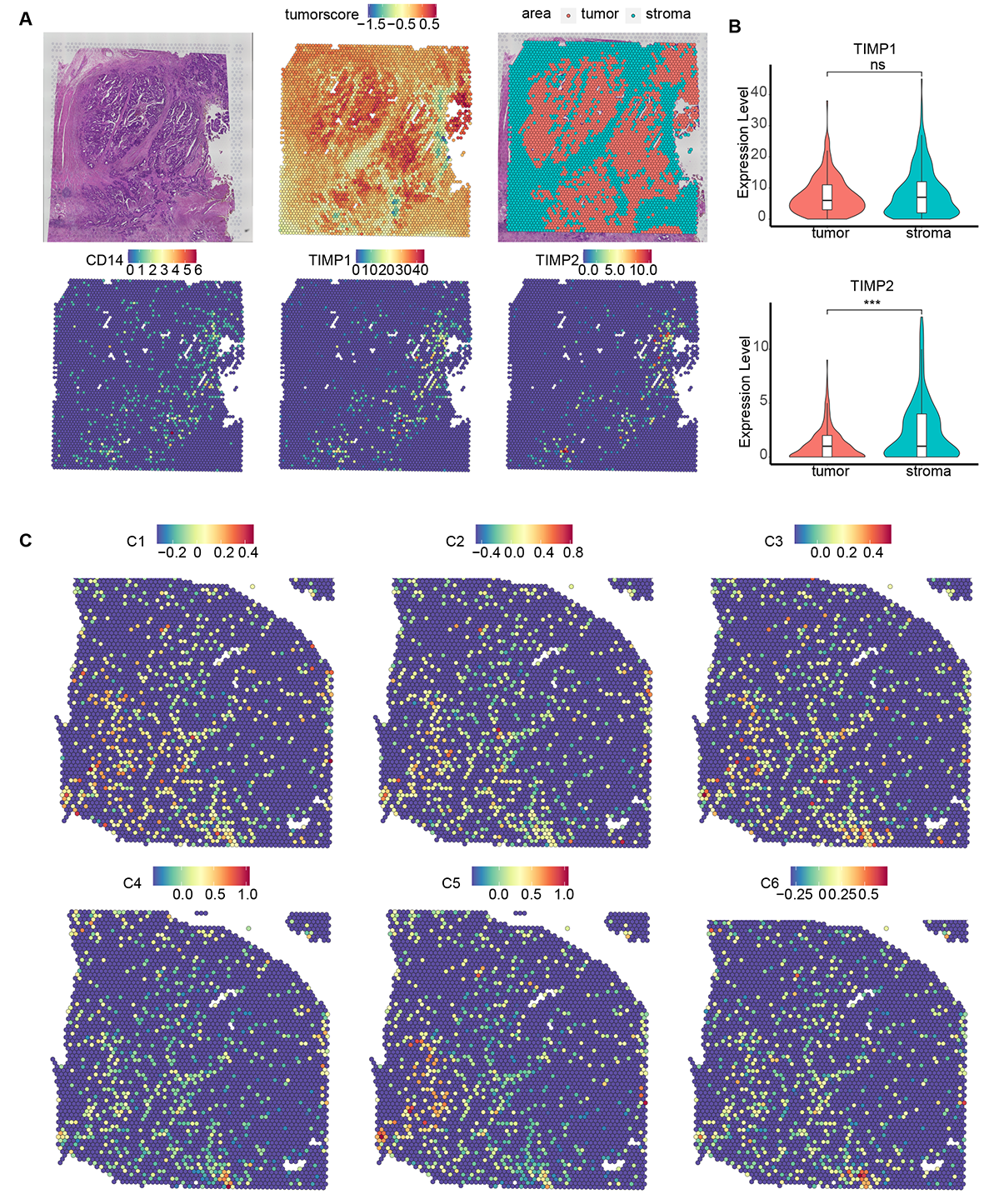

Supplement: S4 Fig — (A). Spatial plots of another CRC sample showed areas of tumor and stroma based on tumor score in tissue sections (top), and distribution signature of TIMP1 and TIMP2 in CD14-expressed area (bottom). (B). Violin plots showed expression level of TIMP1 and TIMP2 in different CD14-expressed areas. (C). Spatial feature plots showed distribution signature of six clusters in tissue sections, respectively. ns represent no significance, * represent p value < 0.05, ** represent p value < 0.01, *** represent p value < 0.001. (TIF) [file pgen.1011176.s004.tif]

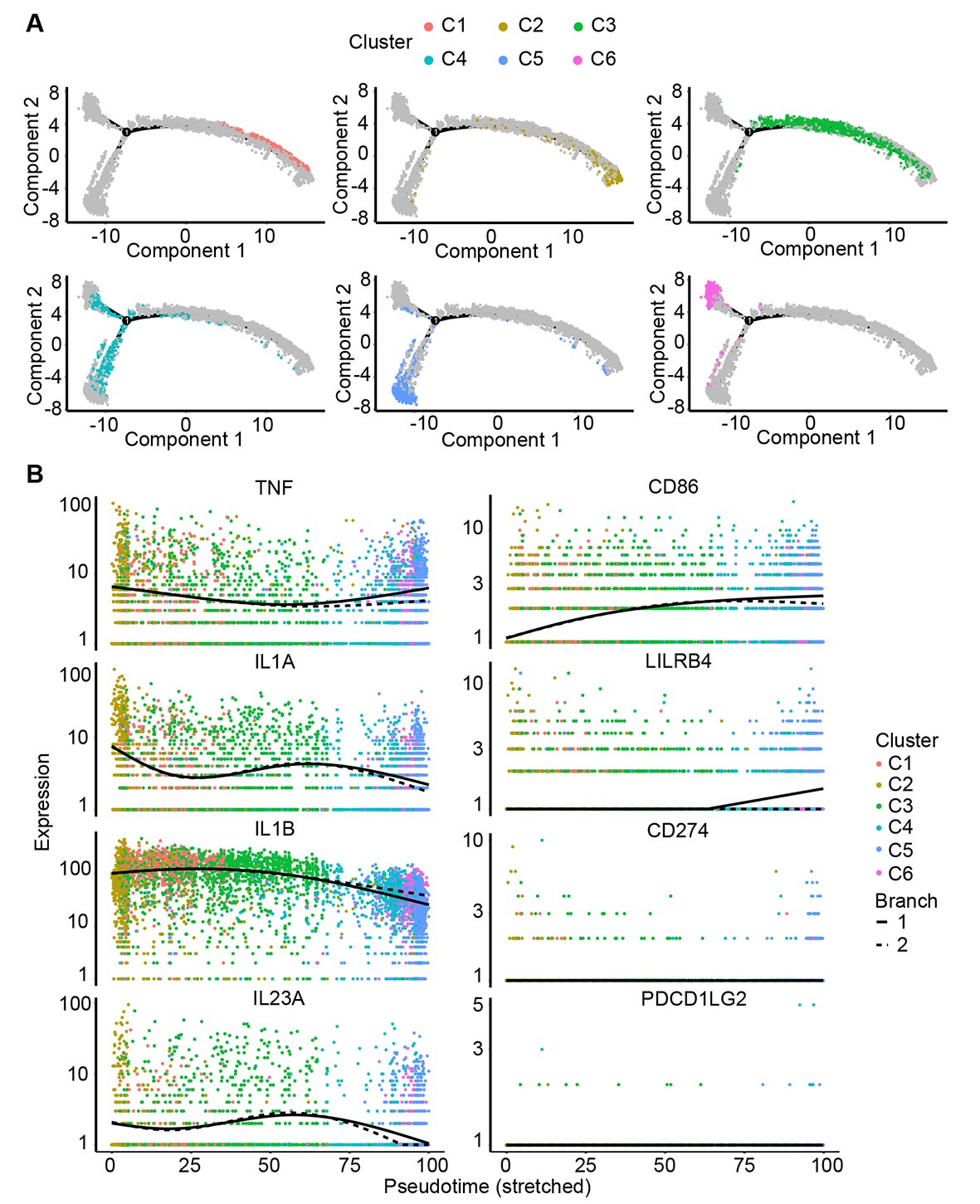

Supplement: S5 Fig — (A). Split pseudotime plot of mononuclear phagocytes showed stages of each cluster. (B). Scatterplots showed downregulated inflammatory genes and upregulated immunosuppression-related genes in the differentiation of mononuclear phagocytes. Cells are colored by clusters. (TIF) [file pgen.1011176.s005.tif]

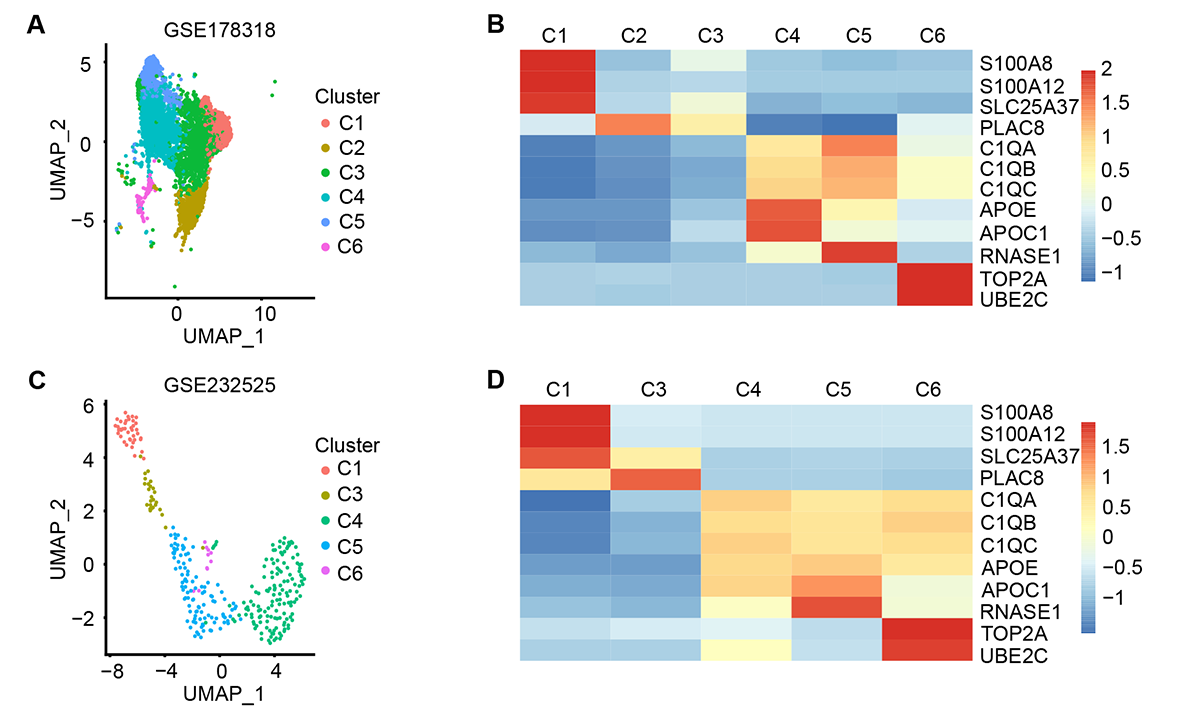

Supplement: S6 Fig — (A). UMAP plot of mononuclear phagocytes in GSE178318 CRC patients. Cells are colored by clusters. (B). Heatmap showed similar expression characteristics of marker genes between GSE178318 and our data. (C). UMAP plot of mononuclear phagocytes in GSE232525 CRC patients. Cells are colored by clusters. (D). Heatmap showed similar expression characteristics of marker genes between GSE232525 and our data. (TIF) [file pgen.1011176.s006.tif]

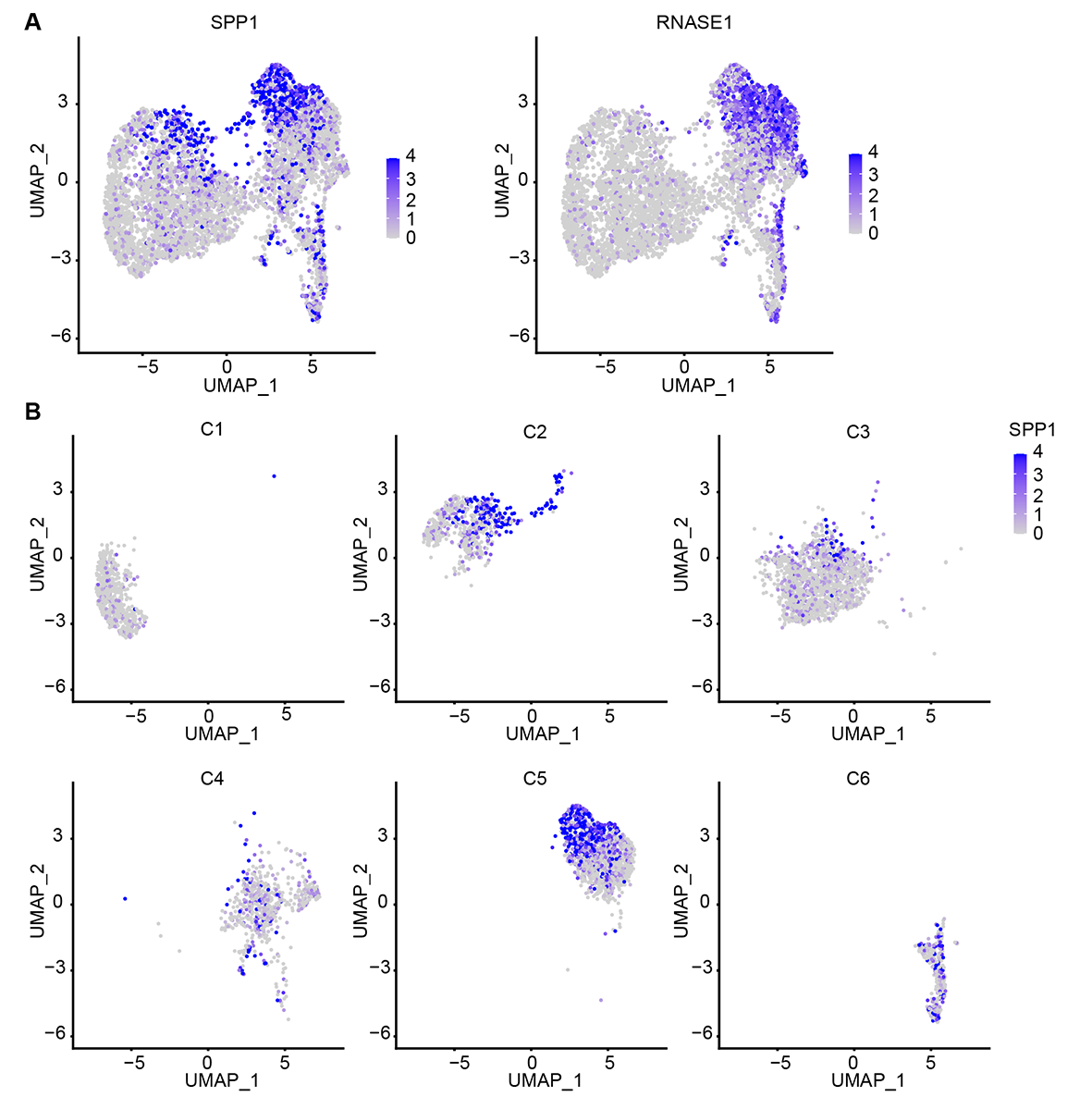

Supplement: S7 Fig — (A). UMAP plot showed expression level of SPP1 and RNASE1 in MPS. (B). Split UMAP plots showed expression levels of SPP1 in each cluster. (TIF) [file pgen.1011176.s007.tif]

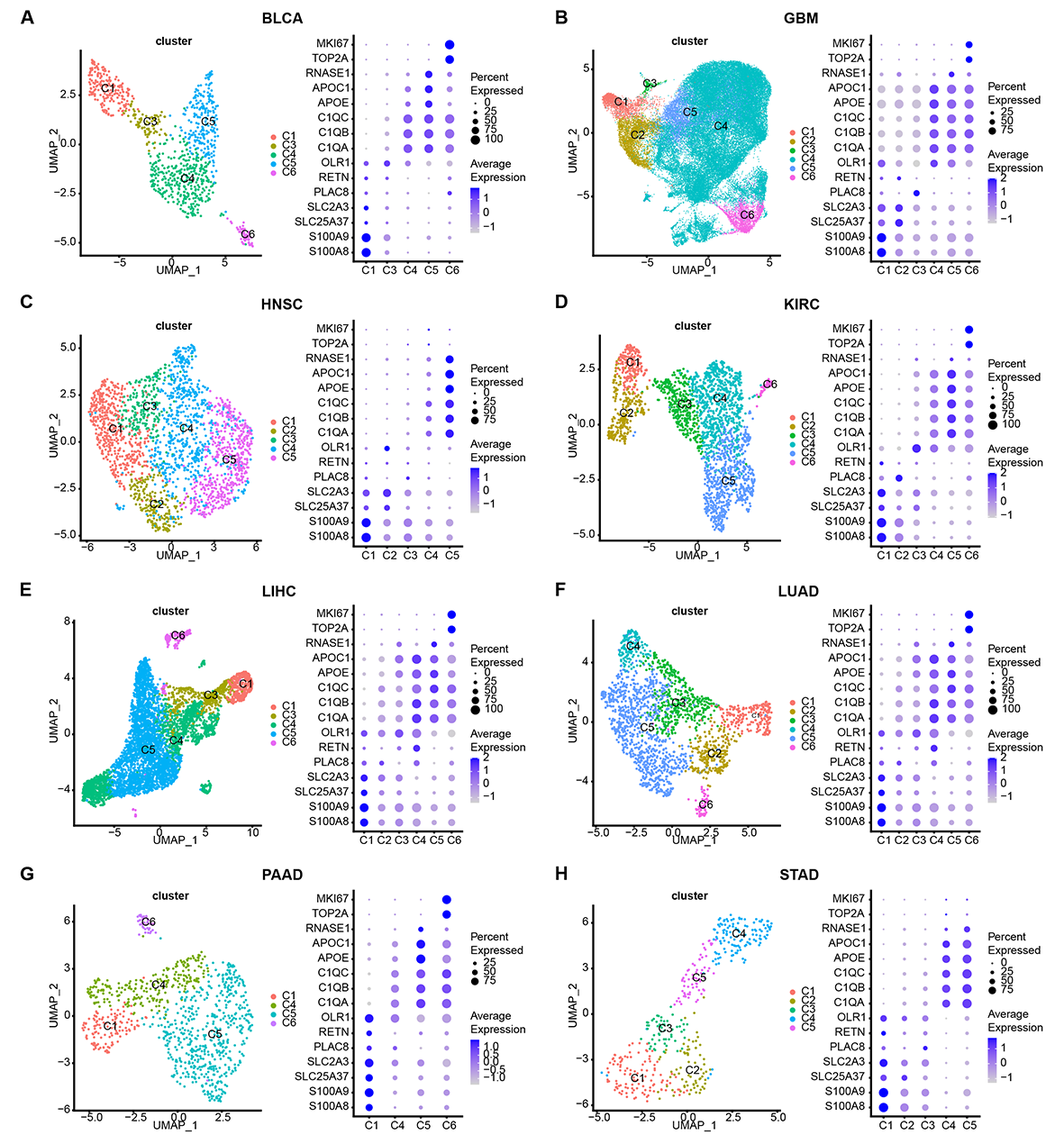

Supplement: S8 Fig — (A-H). UMAP plot (left) and dot plot (right) showed mononuclear phagocytes subsets in BLCA (A), GBM (B), HNSC (C), KIRC (D), LIHC (E), LUAD (F), PAAD (G) and STAD (H). (TIF) [file pgen.1011176.s008.tif]
