# Supplementary material for: Single-cell RNA profiling reveals classification and characteristics of mononuclear phagocytes in colorectal cancer
Source: PLoS Genet. 2024 Feb 26;20(2):e1011176. doi: 10.1371/journal.pgen.1011176 (PMC10919852; doi:10.1371/journal.pgen.1011176)
Supplement: Attachment — Submitted filename: The respones to the reviewers--1231.docx [file pgen.1011176.s009.docx]

**Responses to the reviewer’s comments:**

We thank the Reviewers for their extensive comments and we are very pleased that the reviewers think our manuscript has several positives. We believe their comments have helped us substantiate our conclusions and strengthen our manuscript. Please see below a detailed point-by-point response to all comment.

All changes are highlighted in red in the revised manuscript. Reviewers’ comments are responded point-by-point as below and our responses are highlighted in blue.

Reviewer #1: Thank you for the opportunity to review “Single-cell RNA profiling reveals classification and characteristics of mononuclear phagocytes in colorectal cancer”. The study builds on current understanding in the field that tumor-associated macrophages in colorectal cancer have properties that can drive tumorigenesis. The focus of the current study examines gene expression of CD14-enriched populations from colon tumors excised from three patients. Six clusters are identified and are suggested to arise from developmental progression in order from 1-6. One cluster, C5, is selected for further analysis and two genes enriched in these cells (APOE and RNASE1) are shown to be linked to poor prognosis in CRC. Additionally, using software that infers the abundance of specific cell types among datasets of publicly available bulk-RNAseq, the increased abundance of the C5 cluster is inferred to be associated with poor survival probably in other cancers. While there are some suggestive findings in the present study, the conclusions are not well supported by the data shown. It is also unclear that the findings significantly build upon current understanding in the field. Moreover, there are a number of concerns that bring into question the identities of the cell types examined.

Major concerns are as follows:

The populations studied are enriched by CD14-bead selection, but the data are not examined for the inclusion of CD14-low populations, which would include neutrophils. Neutrophils express the S100 family of proteins and the possibility that neutrophils are present among the clusters examined is not excluded. Also, in some cases, CD14 is expressed by dendritic cells and the dataset is not evaluated to ensure that dendritic cells are excluded. Dendritic cells are a constituent of the mononuclear phagocyte population and the data should be explicit about whether or not these are present—as written, the study would suggest that only macrophages and monocytes are components of the mononuclear phagocyte network, which is incorrect. An evaluation of CSF1R is performed, and it is proposed that these are exclusively expressed by only some clusters. This would seem in contrast with current literature indicating that mononuclear phagocytes all express CSF1R to some respect, and can be further upregulated in pathologic settings (Combes, Immunother Adv, 2021). The authors should clarify the criteria set for CSF1R positive and negative populations. The negative populations may not belong to the mononuclear phagocyte network.

**Response:** Thanks for the reviewer’s advice. The cells sorted were used to perform scRNA-seq. After quality control and removal of the batch effect, Cell-type annotation was performed based on R package SingleR (version 1.8.1). Monocytes and macrophages were selected as the research object. Finally, 5153 cells were remained for downstream analyses. All cells were confirmed to be MPS, with marker gene of mononuclear phagocytes (CD14) highly expressed and marker genes of intestinal dendritic cells (CXCR1) and neutrophils (MPO and CEACAM8, also named CD66b) rarely expressed (Fig. R1). Taken together, the cells we analyzed can be identified as mononuclear phagocytes.

We have now added this data to Supplementary Figure 1C and added the following text to the results sections of the manuscript:

*All cells were confirmed to be MPS, with marker gene of mononuclear phagocytes (CD14) highly expressed and marker genes of intestinal dendritic cells (CX3CR1) and neutrophils (MPO and CEACAM8, also named CD66b) rarely expressed (S1C Fig) [13-15].Then these cells were clustered into six clusters: cluster 1 (C1) to cluster 6 (C6).*

In addition, according to your suggestions, we have modified the evaluation of CSF1R，We evaluated markers of currently known macrophage subdivisions (M1, M2a, M2b, M2c and M2d)，markers of these subdivisions could not distinguish MPS in CRC (Fig. R2). Therefore, we believe that more research on the subpopulation of macrophages is necessary. We have now added this data to Figure 3A and added the following text to the results sections of the manuscript:

*Further studies found, according to different stimuli, the M2* *macrophages can be subdivided to M2a, M2b, M2c and M2d macrophages [21]. However, markers of these subdivisions [6, 22, 23] could not distinguish MPS in CRC (Fig 3A).*

Figure R1 (A). UMAP plots showed expression levels of marker genes of mononuclear phagocytes (CD14), intestinal dendritic cells (CX3CR1) and neutrophils (MPO and CEACAM8, also named CD66b).

Figure R2 (A). UMAP plots showed expression levels of M1 markers (TLR2, TLR4), M2a markers (MRC1, CD209), M2b markers (CD86, CCL1), M2c markers (CD163, MERTK) and M2d markers (VEGFA, IL10).

Another major concern is the absence of an attempt to align the clusters found in the current study with mononuclear phagocytes (including those which are CD14+) known to reside in the healthy intestine. These would include analyses to designate populations distinguished by standard criteria of expression of CD16, HLA-DR, CD11b, CD88, CD64, CD1c and other standard criteria used to subset intestine-resident mononuclear phagocytes (described in many publications, including Bujko, J exp med, 2018; Garrido-Trigo, Nat Comm, 2023, Hegarty, Nat Review Gastro and Hepato, 2023). This will fortify the data in specifying which populations are monocytes, transitioning monocytes and other developmental fates as the authors suggest. Whether or not these standard criteria can be applied to the dataset, there is still a need to understand whether the clusters described in the current report exist in the healthy intestine, which can be ascertained from analysis of publicly available datasets.

**Response:** The reviewer raises an important point. To explore whether the clusters described in the report exist in the healthy intestine, public scRNA-seq datasets of blood from healthy people (GSE167363), colon from healthy people (GSE214695), blood and normal colon from CRC patients (GSE161277) were used for bioinformatics Analysis (fig R3). we have added the following to the results section:

*In healthy intestinal tissue, MPS consists of blood-derived monocytes and tissue-resident macrophages [20]. To explore the origins of the identified MPS subsets in CRC, we aligned our data with data of blood and normal colon tissue from healthy people and CRC patients. The integrated data showed similar clusters of MPS (Fig 2A, B), while proportion of each cluster varied in different sample and tissue (Fig 2C, D). The main MPS cluster in blood was C1 and blood-derived monocyte marker (S100A8) [20] was highly expressed in blood data (Fig 2E), indicating C1 as blood-derived monocytes. In normal colon, C1 and C5 were main MPS clusters and mucosal macrophages marker (SELENOP) [20] was highly expressed in C5 (Fig 2E), suggesting that C5 was tissue-resident macrophage. Compared with healthy people, normal colon in CRC patient showed a higher proportion of C1. MPS in cancer tissue showed completely different clustering, with proportion of C2 increased and C3, C6 specifically appeared in cancer tissue. These results indicated tumor microenvironment of CRC would result in unique polarization of MPS.*

Figure R3 (A). UMAP plot of mononuclear phagocytes from blood, normal colon and CRC from healthy and CRC samples. (B). Dot plot showed marker genes of six clusters. (C and D). Split UMAP plot (C) of mononuclear phagocytes and bar chart (D) showed proportion of six clusters in different sample. (E). Split UMAP plots showed expression levels of markers of intestinal monocytes (S100A8) and mucosal macrophages (SELENOP) in mononuclear phagocytes.

Currently, the authors do not fully describe the clusters. Gene expression the authors put forward to distinguish clusters is not rigorously tested and/or attempted to align with published literature on those genes, as they are expressed by macrophages. Given that there is existing literature on several of the cluster-defining genes reported here, the authors need to give more attention to previous reports to expand on current understanding. The authors should attempt to stain for more cluster-defining genes in tissue samples. The authors should also investigate whether gene expression of other similar genes align with their findings, such as the author’s claims for higher phagocytic potential. Information on other gene expression for complement genes would also be of interest to the field. To further address the study’s focus on macrophage heterogeneity, the authors should align and discuss their findings as compared to the published literature on M2-like clusters C4 to C6 with the M2a, M2b, M2c, and M2d stratificaitons previously reported. An effort to align results with that which is already known will advance the field in regards to better understanding of TAMs subsets.

**Response:** Thanks for the reviewer’s advice. We stained six clusters by immunofluorescence, as shown in the figure (Fig. R4), and these subpopulations are indeed present in human colon cancer tissue. We have now added this data to Supplementary Figure 2B of the manuscript.

Figure R4 (A). Immunofluorescence staining of CD14 and marker genes of each cluster in CRC tissues confirmed the existence of six clusters. White arrows point cells of each cluster. Scale bars, 20 μm.

To address the reviewer’s advice, we describe each new subpopulations in more detail (Fig R5). We have added the following to the results section:

*Functional analysis showed different functional characteristics in each cluster (Fig 1E). C1, C2 and C3 were enriched in pathogen-associated molecular patterns (PAMP) receptor signaling pathway, indicating function of innate immunity [18]. While C4, C5 and C6 showed an enrichment in antigen processing and presentation, indicating their role in adaptive immunity [19]. Besides, C1 was enriched in monocyte aggregation, C5 had ability to clear lipoprotein particle, and C6 showed a stronger cell cycle DNA replication.*

Figure R5 (A). Radar charts showed GSVA score of biological process GO term in six clusters.

In addition, according to your suggestion, we evaluated markers of currently known macrophage subdivisions (M1, M2a, M2b, M2c and M2d)，The results showed that markers of these subdivisions could not distinguish MPS in CRC (Fig R2).

There is a need to describe the patients and how these differences might align with differences in the cluster abundance. For example, C5 was the dominant TAMs in WUH1, while WUH2 and WUH3 patients have more abundant M1-like macrophage clusters. Could the author please comment whether the tumor stages or progression of those three patients as they potentially affect the TAMs population distribution?

**Response:** We thank the Reviewer for this suggestion. We describe the information of three CRC patients in Table 1.

Table 1

Preoperative and pathologic characteristics of CRC patients

|  | WHU1 | WHU2 | WHU3 |
| --- | --- | --- | --- |
| Gender | female | male | male |
| Age at diagnosis | 70 | 66 | 76 |
| Site of tumor | Ascending colon | rectum | descending colon |
| Time of surgery | 2020/12/14 | 2021/1/18 | 2019/11/26 |
| Pathology | moderately differentiated adenocarcinoma | moderately poorly differentiated adenocarcinoma | moderately differentiated adenocarcinoma |
| Stage | Ⅱ | Ⅲ | Ⅱ |
| T | 3 | 3 | 3 |
| N | 0 | 1 | 0 |
| M | 0 | 0 | 0 |
| preoperative chemotherapy | No | No | No |

The pathology of three patients were moderately differentiated adenocarcinoma, moderately poorly differentiated adenocarcinoma, and moderately differentiated adenocarcinoma. The TNM stages are: T3N0M0, T3N1M0, T3N0M0. We also analyzed the distribution of TAM subgroups in the tumor tissues of colon cancer patients at different stages. As shown in (Fig. R6A), all CRC patients had C1-C6 subpopulations, among which the proportion of C5 showed a trend of decreasing first and then increasing, while the proportion of C6 subpopulation gradually decreased.

Figure R6 (A). Bar charts showed showed proportion of six clusters in different tumor stage.

The authors refer to the C5 cluster as immunosuppressive, but provide no functional data to support that conclusion. Since C4 and C6 also shows M2-like macrophage phenotype, it will be important for the authors to show whether C4 or C6 cluster (and all the other clusters) correlated with poor prognosis as well. This is particularly important because C4 shares a lot of features with C5, including the TF regulons reported identified by the authors. Moreover, the author’s focus on C5 is unclear, given that the data shown suggests the C4 population is both more abundant and responsive to PC treatment (Fig 5).

**Response:** To address the reviewer’s advice and explore the relationship between MPS subsets with CRC prognosis, we correlated our scRNA-seq data to public datasets. Abundance of each cluster in tumor tissue was evaluated with CYBERSORTx. Results showed only C5 is associated with worse prognosis in CRC (Fig R7). We have now added this data to Figure 3A and added the following text to the results sections of the manuscript:

*To explore the relationship between MPS subsets with CRC prognosis, we correlated our scRNA-seq data to public datasets. Abundance of each cluster in tumor tissue was evaluated with CYBERSORTx, a computational framework for inferring cell type abundance and cell-type-specific transcriptomes from RNA profiles of intact tissue [27]. Results showed only C5 is associated with worse prognosis in CRC (Fig 7A).*

Figure R7 (A). Kaplan–Meier survival curves of TCGA COAD and READ patients grouped by abundance of six mononuclear phagocytes subsets.

Fig 5: The authors analyzed scRNAseq dataset from preoperative chemotherapy (PC) treated patients and showed C5 was significantly reduced with tumor shrinkage. The caveat that PC has direct effect to diminish population abundance of C5 is not addressed. Can the authors provide further validation of their conclusions by analyzing other publicly available datasets from CRC patients receiving other methods of treatment?

**Response:** To address the reviewer’s advice, CRC patients treated with or without preoperative chemotherapy (PC) (GSE178318) and CRC patients before and after chemotherapy (GSE232525) were used for bioinformatics Analysis. PC significantly reduced C5 abundance in CRC (Fig R8A, B). CRC before and after chemotherapy showed similar results (Fig R8C, D). We have now added this data to Figure 6A-D. The following text have added to the results sections of the manuscript:

*To explore the role of C5 in tumor treatment, we analyzed publicly available scRNA-seq dataset from CRC patients treated with or without preoperative chemotherapy (PC) (GSE178318) and CRC patients before and after chemotherapy (GSE232525). The public scRNA-seq dataset showed similar clusters of mononuclear phagocytes with our data (S6A-D Fig). PC significantly reduced C5 abundance in CRC (Fig 6A, B). CRC before and after chemotherapy showed similar results (Fig 6C, D). These patients who have undergone treatment showed significant tumor shrinkage, suggesting that C5 is a potential target cluster for CRC treatment.*

Figure R8 (A). UMAP plots of mononuclear phagocytes in CRC with treatment-naïve (left) and treated with PC (right). Cells are colored by clusters. (B). Bar plot showed proportion of different clusters in CRC with treatment-naïve and treated with PC. (C). UMAP plots of mononuclear phagocytes in CRC before chemotherapy (left) and after chemotherapy (right). Cells are colored by clusters. (D). Bar plot showed proportion of different clusters in CRC before and after chemotherapy.

Fig 2f: Conclusions of the imaging data are unclear, given that there is limited overlap of stains with CD14. There is a need for quantification to support the author’s conclusions, as well as additional samples tested.

**Response:** Thank you for your advice. We analyzed and quantified the spatial transcriptome data in more detail. As the result shown, MPS with high expression of TIMP1 were distributed more within the tumor, while MPS with high expression of TIMP2 were distributed more in stroma (Fig R9 and R10A, B). Besides, spatial distribution of different clusters varied as well (Fig R10C). We have now added this data to Figure 3F, G and Supplementary Figure 4A-C. The following text have added to the results sections of the manuscript:

*To decipher the spatial distribution of mononuclear phagocytes with high expression of TIMP1 or TIMP2 in the tumor microenvironment, spatial transcriptome data of CRC was analyzed. Tumor area was identified by tumor score and CD14 was used to label mononuclear phagocytes. As the result shown, MPS with high expression of TIMP1 were distributed more within the tumor, while MPS with high expression of TIMP2 were distributed more in stroma (Fig 3F, G and S4A, B Fig). Besides, spatial distribution of different clusters varied as well (S4C Fig).*

Figure R9 (A). Spatial plots showed areas of tumor and stroma based on tumor score in tissue sections (top), and distribution signature of TIMP1 and TIMP2 in CD14-expressed area (bottom). (B). Violin plots showed expression level of TIMP1 and TIMP2 in different CD14-expressed areas. * represent p value < 0.05, ** represent p value < 0.01, *** represent p value < 0.001.

Figure R10 (A). Spatial plots of another CRC sample showed areas of tumor and stroma based on tumor score in tissue sections (top), and distribution signature of TIMP1 and TIMP2 in CD14-expressed area (bottom). (B). Violin plots showed expression level of TIMP1 and TIMP2 in different CD14-expressed areas. (C). Spatial feature plots showed distribution signature of six clusters in tissue sections, respectively. ns represent no significance, * represent p value < 0.05, ** represent p value < 0.01, *** represent p value < 0.001.

The author’s suggestion that the C6 cluster is proliferative may indicate the need to perform a second pseudotime analysis without this population being considered. Proliferation of mature macrophages may indeed be altered in the tumor environment (Soncin, Nature Communications, 2018), and the author’s study is an opportunity to further test this hypothesis. Another hypothesis to be tested would be that the proliferating populations are not dependent on monocyte progenitors and thus, inappropriately placed on the pseudotime analysis. These hypotheses should be tested in this report.

**Response:** We thank the reviewer for this suggestion. In healthy intestinal tissue, MPS consists of blood-derived monocytes and tissue-resident macrophages. To explore the origins of the identified MPS subsets in CRC, we aligned our data with data of blood and normal colon tissue from healthy people and CRC patients. Our results show that, consistent with Soncin (Nature Communications, 2018), the proliferative subpopulation C6 is only present in tumor tissue (Fig R3). Therefore, we think that C6 may be differentiated from peripheral blood origin monocytes, so C6 should be placed on the pseudotime analysis.

M1 and M2 macrophages are both terminally differentiated subsets. Although the authors found different expression pattern of TIMP1 and TIMP2 in M1-like and M2-like macrophages, the analysis in figure 2 did not support the idea that TIMP1 and TIMP2 are involved in mononuclear phagocytes differentiation or M1 to M2 transition plasticity. Especially, by the trajectory and RNA velocity analysis, the authors claimed a development pathway from C1 to C5, however, the expression pattern of TIMP1 and TIMP2 was varied among clusters, which did not support the trajectory. The authors should consider the use of human monocyte-derived cultures to test their hypotheses.

**Response:** We thank the reviewer for this suggestion. To address this, human blood-derived monocytes were used to verify our results. Q-PCR analysis showed that, compared with M2 macrophages, M1 macrophages expressed higher TIMP1 and lower TIMP2 (Fig R11). We have now added this data to Supplementary Figure 3F and added the following text to the results section of the manuscript:

*To further verify this finding, we obtained human blood-derived monocytes and induced them into M1 or M2 macrophages. Q-PCR analysis showed that, compared with M2 macrophages, M1 macrophages expressed higher TIMP1 and lower TIMP2 (S3F Fig).*

Figure R11 (A). Expression levels of TIMP1 and TIMP2 in human monocyte-derived M1 and M2 cells (N=3), *P < 0.05. The statistical significance was obtained by Student's t-tests.

The authors group CD86 as a immunosuppressive gene, but this is not the typical classification for the CD86 T cell co-stimulatory pathway, and the citation for the author’s functional annotation for CD86 as immunosuppressive in colorectal cancer is not described. By contrast, while CD274’s role in T cell exhaustion is well known, CD274’s role in colorectal cancer is not well established. Since CD274 is naturally expressed by intestinal macrophages in conditions of health, the authors should provide citations to expand on the understanding of the roles of CD274 in colorectal cancer as they are interpreting it in this study.

**Response:** Thank you very much for your suggestions. We now added the citations of the studies of CD274.

*During the differentiation of mononuclear phagocytes, the* *expression of inflammatory genes (TNF, IL1A, IL1B, IL23A) and* *immunosuppression-related genes (CD86, LILRB4, CD274 [25] PDCD1LG2) was gradua**lly decreased and increased, respectively (Fig 4D, S5B Fig).*

References:

*[25]* *Pardoll DM. The blockade of immune checkpoints in cancer immunotherapy. Nat Rev Cancer. 2012;12(4):252-264.*

In this paper, the author showed that CD274, as a ligand of PD1, can form an immunosuppressive microenvironment by inhibiting T cell immune function.

Fig 6: The author’s comparisons of C5 versus non-C5 is inappropriate, given that the non-C5 are heterogenous. Comparisons to each individual cluster would be more informative for the proposed differences. Also, GO terms should be provided for all the clusters to best understand their distinctions.

**Response:** We thank the reviewer for this suggestion. we have compared each individual cluster. Functional analysis showed different functional characteristics in each cluster (Fig R5). C1, C2 and C3 were enriched in pathogen-associated molecular patterns (PAMP) receptor signaling pathway, indicating function of innate immunity. While C4, C5 and C6 showed an enrichment in antigen processing and presentation, indicating their role in adaptive immunity. Besides, C1 was enriched in monocyte aggregation, C5 had ability to clear lipoprotein particle, and C6 showed a stronger cell cycle DNA replication.

Fig S5. The author’s conclusions that the C5 cluster is distinct from SPP1+ macrophages is unclear, given that the data shows clear overlap between SPP1 expression and the C5 cluster. There appears to be some alignment, which can be discussed in the results appropriately.

**Response:** Some studies also identified SPP^+^ macrophages as a pro-tumorigenic/ pro-metastatic cluster in CRC, but doubts arose as to whether C5 were the same as SPP^+^ macrophages. Our study found that the comparison of gene signature between C5 and SPP1^+^ macrophages showed SPP1 was highly expressed in part of C2, C5 and C6. These results revealed a clear difference between C5 and SPP1^+^ macrophages, but the relationship between these two cell clusters needs further research.

Fig7: The authors need to clarify the criteria used to establish “mononuclear phagocytes” and “non mononuclear phagocyte” for the APOE and RNASE1 analysis. The authors should also specify whether the “non mononuclear phagocytes” are immune cells, which would be the appropriate population comparison. A comparison of the tumor expression would also be informative.

**Response:** We thank the Reviewer for this suggestion. We analyzed the expression level of APOE and RNASE1 in mononuclear phagocytes, B cell, DC, Neutrophils, NK cell, T cell, Endothelial cell and fibroblasts. The public scRNA-seq dataset of CRC showed that APOE and RNASE1, two marker genes of C5, were highly co-expressed in mononuclear phagocytes only (Fig R12). We have now added this data to Figure 7B and added the following text to the results section of the manuscript:

*The public scRNA-seq dataset of CRC showed that APOE and RNASE1, two marker genes of C5, were highly co-expressed in mononuclear phagocytes only (Fig 7B).*

Figure R12 (A). Violin plots showed expression levels of APOE (top) and RNASE1 (bottom) in different types of cells.

Last, the APOE and RNASE1 expression in CRC described here is interesting. For their conclusions beyond CRC, the data are suggestive but far from conclusive. While the existence of a “universal” tumor-associated macrophage associated with poor prognosis in solid tumors would be of interest, there is not enough data to support the authors claims. Intestine is known to have unique populations of mononuclear phagocytes, and it cannot be assumed that these are present (or of similar identity) in non-intestinal tissues. Moreover, gene expression that distinguishes the different populations of mononuclear phagocytes is well known to be tissue specific and assumptions of identity (without further validation) should be minimal. There needs to be transparent criteria of matching for the C5 cluster and transparency of criteria sorting into high vs. low abundance of the C5 cluster, and whether it is found in all datasets evaluated, to determine whether the cluster is of relevant amounts to be considered physiologically relevant in non-intestinal tissues.

**Response:** To address the reviewer’s advice, BLCA patients (GSE190888), GBM patients (GSE162631), HNSC patients (GSE234933), KIRC patients (GSE121636), LIHC patients (GSE242889), LUSC patients (GSE123904), PAAD patients (GSE214295), STAD patients (GSE234129) were used for bioinformatics Analysis. MPS in these tumors had similar clusters with C5 present in all tumors (Fig R13). Survival analysis showed C5 was associated with a worse prognosis of patients with BLCA, GBM, HNSC, PAAD and STAD, with no significance in LUSC patients, and was associated with better prognosis in KIRC and LIHC (Fig R14). We have now added this data to Supplementary Figure 8A-H and Figure 7D. The following text have added to the results section of the manuscript:

*To expand these observations to more tumors, we explored scRNA-seq datasets of* *bladder cancer (BLCA), glioblastomas (GBM), head and neck cancer (HNSC), kidney clear cell cancer (KIRC), liver cancer (LIHC), lung cancer (LUSC), pancreatic cancer (PAAD) and gastric cancer (STAD). The results showed that MPS in these tumors had similar clusters with C5 present in all tumors (S8A-H Fig). Survival analysis showed C5 was associated with a worse prognosis of patients with BLCA, GBM, HNSC, PAAD and STAD, with no significance in LUSC patients, and was associated with better prognosis in KIRC and LIHC (Fig 7D).*

Figure R13 (A-H). UMAP plot (left) and dot plot (right) showed mononuclear phagocytes subsets in BLCA (A), GBM (B), HNSC (C), KIRC (C), LIHC (D), LUSC (E), PAAD (F) and STAD (G).

Figure R14 (A). Kaplan–Meier survival curves of TCGA BLCA, GBM, HNSC, KIRC, LIHC, LUSC, PAAD and STAD patients grouped by C5 abundance. P values were calculated using the log-rank test.

In line 113, the author claimed that in WUH3 patient had a higher portion of C2 but by figure 1E, the most abundant population in WUH3 was C3.

**Response:** Thank you very much for your helpful suggestions. We have double-checked all the manuscript to avoid similar mistakes.

Reviewer #2: I have read with interest this study that concerns the classifications of different populations of TAMS in colorectal cancer. By using the scRNAseq technology, the authors reported 6 different TAMs subsets in colorectal cancer showing, in particular, that there was an immunosuppressive subset associated with worse survival.

I believe that this manuscript is overall well-written, and it might be of interest to the readership of PLOS Genetics. However, I would suggest the following changes:

1. The clinical data of the three CRC patients should be added. General clinical, pathological, and surgical data should be given.

**Response:** We thank the reviewer for this suggestion. We describe the clinical data of three CRC patients in Table 1.

Table 1

Preoperative and pathologic characteristics of CRC patients

|  | WHU1 | WHU2 | WHU3 |
| --- | --- | --- | --- |
| Gender | female | male | male |
| Age at diagnosis | 70 | 66 | 76 |
| Site of tumor | Ascending colon | rectum | descending colon |
| Time of surgery | 2020/12/14 | 2021/1/18 | 2019/11/26 |
| Pathology | moderately differentiated adenocarcinoma | moderately poorly differentiated adenocarcinoma | moderately differentiated adenocarcinoma |
| Stage | Ⅱ | Ⅲ | Ⅱ |
| T | 3 | 3 | 3 |
| N | 0 | 1 | 0 |
| M | 0 | 0 | 0 |
| preoperative chemotherapy | No | No | No |

2. Which were the TNM stages?

**Response:** As shown in Table 1. The TNM stages of three patients are: T3N0M0 (WHU1), T3N1M0 (WHU2), T3N0M0 (WHU3).

3. Were these 3 patients homogenous? Had they similar clinical and pathological characteristics?

**Response:** The pathology of three patients were moderately differentiated adenocarcinoma, moderately poorly differentiated adenocarcinoma, and moderately differentiated adenocarcinoma. Well-differentiated adenocarcinomas were not included in the current case, and the similar clinical characteristics was that none of the three patients received preoperative chemotherapy.

4. Did they get chemotherapy before colectomy?

**Response:** None of the three patients received preoperative chemotherapy.

5. The protocol of specimen retrieval should be given. From which part of the colon were the specimen taken? Directly from the tumor o from the peri-tumoral areas?

**Response:** We thank the reviewer for this suggestion. In this study, we directly took tumor tissues from surgical specimens (1*1*1 cm) of three CRC patients to prepare single-cell suspension and then performed scRNA-seq. The following text have added to the methods section of the manuscript:

*Three patients (WHU1, WHU2, WHU3) were pathologically diagnosed CRC and specimens (1*1*1 cm) were took directly from the tumor and collected for scRNA-seq. Clinical information of patients were included in table 1.*

6. The following papers should be considered and eventually added / commented in the discussion: 1) Cortese N, et al. Cancer Immunol Res. 2023 Apr 3;11(4):405-420; 2) Donadon M, et al. J Exp Med. 2020 Nov 2;217(11):e20191847; 3) Murray PJ. J Exp Med. 2020 Nov 2;217(11):e20201259).

**Response:** Thank you for your suggestion. In the discussion section, references including Cortese N, et al. and Donadon M, et al. are cited and discussed.

*In exploring the origins of the identified MPS subsets in CRC，we found that C1 was blood-derived monocyte and mucosal macrophages marker (SELENOP) was highly expressed in C5, suggesting that C5 was tissue-resident macrophage. Immunosuppressive cluster C5, which highly expresses C1QA, APOE and RNASE1, was at the terminal state of differentiation and played an important role in the formation of an immunosuppressive tumor microenvironm**ent [43,44].*

*Cortese N, et al. have reported that GPNMB+ TAM is negatively associated with CRC prognosis. GPNMB^+^ TAM more often accumulated at the tumor margin, therefore more exposed to tumor immunosuppressive signals, favor tumor growth and metastasis [45]. In our study, C5 was more accumulated at the tumor margin. Whether the C5 subset can promote CRC metastasis remains to be explored.*

7. The authors should investigate more the relationship between TIMPs, the C5 subset and the associated lipid metabolism. Since they reported such relationships, a lipidomic approach should be given otherwise it remains a speculation.

**Response:** Thanks to the reviewer for the suggestion. Our results showed that C5 highly expressed APOE, RNASE1, and APOC1, Functional analysis showed C5 showed an enrichment in antigen processing and presentation and had ability to clear lipoprotein particle. ScMetabolism was applied to explore the metabolic characteristics of C5 and found a significant enhancement in lipid metabolism (including Sphingolipid metabolism, Glycosphingolipid biosynthesis, Glycerophospholipid metabolism, Glycerolipid metabolism, Ether lipid metabolism). These results fully illustrate that the lipid metabolism of C5 is different from that of other subpopulations. Due to our lack of suitable surface markers, we were unable to isolate individual subpopulations of macrophages for metabolome assays. In addition, we still lack appropriate in vitro stimulation methods to induce the differentiation of C1-C6, so it is difficult to obtain in vitro induced cells for detection. At this stage, we can only describe the metabolic characteristics of the C5 through scRNA-seq.

8. What is really missing in this paper, and it would be nice to have, is a kind of validation of authors’ scRNAseq data. FACS, PCR and/or IHC analyses would be of added value.

**Response:** Thank you for your advice. To further verify our results, we used human blood-derived monocytes to verify differences in the expression of TIMP1 and TIMP2 in M1 and M2 macrophages. Q-PCR analysis showed that, compared with M2 macrophages, M1 macrophages expressed higher TIMP1 and lower TIMP2 (Fig R11). We have now added this data to Supplementary Figure 3F and added the following text to the results sections of the manuscript:

*To further verify this finding, we obtained human* *blood-derived monocytes and induced them into M1 and M2 macrophages.* *QPCR analysis showed that, compared with M2 macrophages, M1 macrophages expressed higher TIMP1 and lower TIMP2 (S3F Fig).*

Figure R11 (A). Expression levels of TIMP1 and TIMP2 in human monocyte-derived M1 and M2 cells (N=3), *P < 0.05. The statistical significance was obtained by Student's t-tests.

In addition, we stained six clusters by immunofluorescence, as shown in the figure (Fig R4), and these subsets are indeed present in human colon cancer tissue. We have now added this data to Supplementary Figure 2B of the manuscript. And the following text have added to the results section of the manuscript:

*The existences of six clusters were further validated by immunofluorescence staining in CRC (S2B Fig).*

Figure R4 (A). Immunofluorescence staining of CD14 and marker genes of each cluster in CRC tissues confirmed the existence of six clusters. White arrows point cells of each cluster. Scale bars, 20 μm.

Reviewer #3: This is an interesting article that adds to the growing body of knowledge with regards to macrophage/TME biology in the context of solid tumors. Like other papers, they describe and annotate macrophage populations within CRCs using scRNA-Seq. However, the authors make some major claims about the significance of their data which need to be backed up with deeper analysis. The manuscript could be improved by clarifying the following:

1. It appears that the clustering results are highly dependent on resolution parameters in Seurat -- the authors observed clusters C1-C6 but it appears that C1-3 are pretty similar to each other (vs C4-5, and C6). Have the authors assessed the robustness of their clustering? Was Leiden or Louvain clustering used to generate them?

**Response:** We thank the reviewer for these comments. We generated our result using Leiden clustering. To test whether our clustering can distinguish subsets well, according to method of Chaffin M (Nature,2022), we calculated AUC of marker genes. Results showed all clusters have a number of genes with AUC > 0.7 (C1: 49, C2: 10, C3: 9, C4: 21, C5: 194, C6: 603), indicating our clustering can distinguish subsets well.

2. Fig 2A and 2B appear to show opposing results: 2A -- where the triangle points show the mean polarization of each cluster, do not appear to have any significant polarization in M1 and have some difference in M2 polarization. Yet, in 2B, it is clear that M1 and M2-classified cells clearly separate in UMAP space. From Fig 1D it looks like C1-3 are M1 and the rest are M2 but 2A does not reflect that at all. Perhaps the authors can clarify.

**Response:** Thank you for your comments. Polarization score of MPS were calculated according to Sun Y (Cell, 2021). They constructed an M1 polarization score gene set using traditional M1 marker genes. However, consistent with their research, our results (Fig 3A) also showed traditional M1 marker genes can't distinguish human M1-like cells well, which result in the phenomenon in Fig 3B. And this is one of reasons we tried to find more specific markers that could distinguish MPS in human CRC.

3. There seems to be a typo on line 134: "special" should read "spatial". I'd advise the authors to search for this elsewhere in the manuscript.

**Response:** We have carefully proofread the manuscript, corrected errors, and apologize for these errors in the first submitted version.

4. It's not clear to me why the authors used spatial analysis strictly for M1/M2 cells, when it appears that the main novelty of the manuscript is the discovery of additional macrophage clusters C1-C6. The section "Tissue inhibitor of metalloproteinases (TIMPs) participate in the differentiation of M1-like and M2-like mononuclear phagocytes" appears to attempt an alignment between C1-6 clusters with canonical M1/M2 polarization, but then does not follow up with how their new findings are interpreted with the spatial information. How are C1-6 spatially distributed? Considering that marker genes were found in each C1-6 cluster why was spatial analysis not done? The authors performed IF in Fig 5C so they have the capability.

**Response:** Thank you for your important suggestions. According to your suggestion, we used the spatial transcriptome data to analyze the expression of TIMP1 and TIMP2 in tumor samples. As the result shown, MPS with high expression of TIMP1 were distributed more within the tumor, while MPS with high expression of TIMP2 were distributed more in stroma (Fig R9 and FigR10A, B). We also show the spatial distribution of C1-C6 in the manuscript (FigR10C Fig). In addition, we stained six clusters by immunofluorescence, as shown in the figure (Fig R4A), C1-C6 are indeed present in human CRC tissue. We have now added this data to Figure 3F-G, Supplementary Figure 4A-C and Supplementary Figure 2B. The following text have added to the results sections of the manuscript:

*To decipher the spatial distribution of mononuclear phagocytes with high expression of TIMP1 or TIMP2 in the tumor microenvironment, spatial transcriptome data of CRC was analyzed. Tumor area was identified by tumor score and CD14 was used to label mononuclear phagocytes. As the result shown, MPS with high expression of TIMP1 were distributed more within the tumor, while MPS with high expression of TIMP2 were distributed more in stroma (Fig 3F, G and S4A, B Fig). Besides, spatial distribution of different clusters varied as well (S4C Fig).*

*The existences of six clusters were further validated by immunofluorescence staining in CRC (S2B Fig).*

Figure R9 (A). Spatial plots showed areas of tumor and stroma based on tumor score in tissue sections (top), and distribution signature of TIMP1 and TIMP2 in CD14-expressed area (bottom). (B). Violin plots showed expression level of TIMP1 and TIMP2 in different CD14-expressed areas. * represent p value < 0.05, ** represent p value < 0.01, *** represent p value < 0.001.

Figure R10 (A). Spatial plots of another CRC sample showed areas of tumor and stroma based on tumor score in tissue sections (top), and distribution signature of TIMP1 and TIMP2 in CD14-expressed area (bottom). (B). Violin plots showed expression level of TIMP1 and TIMP2 in different CD14-expressed areas. (C). Spatial feature plots showed distribution signature of six clusters in tissue sections, respectively. ns represent no significance, * represent p value < 0.05, ** represent p value < 0.01, *** represent p value < 0.001.

Figure R4 (A). Immunofluorescence staining of CD14 and marker genes of each cluster in CRC tissues confirmed the existence of six clusters. White arrows point cells of each cluster. Scale bars, 20 μm.

5. The authors should perform KM-curve analysis using marker genes of their discovered C1-6 clusters, otherwise it's not convincing that C5 itself has a worse prognosis. Otherwise, you could argue that any markers associated with macrophages lead to a worse prognosis.

**Response:** We thank the reviewer for this important suggestion. To address the reviewer’s advice, we correlated our scRNA-seq data to public datasets. Abundance of each cluster in tumor tissue was evaluated with CYBERSORTx. KM-curve analysis showed only C5 is associated with worse prognosis in CRC (Fig R7A). We have now added this data to Figure 3A and added the following text to the results sections of the manuscript:

*To explore the relationship between MPS subsets with CRC prognosis, we correlated our scRNA-seq data to public datasets. Abundance of each cluster in tumor tissue was evaluated with CYBERSORTx, a computational framework for inferring cell type abundance and cell-type-specific transcriptomes from RNA profiles of intact tissue [27]. Results showed only C5 is associated with worse prognosis in CRC (Fig 7A).*

Figure R7 (A). Kaplan–Meier survival curves of TCGA COAD and READ patients grouped by abundance of six mononuclear phagocytes subsets.
